# Neuronal hypofunction and network dysfunction in a mouse model at early stage of tauopathy

**DOI:** 10.1002/alz.089673

**Published:** 2025-01-03

**Authors:** Changyi Ji, Xiaofeng Yang, Yan Lin, Mohamed Eleish, Einar M. Sigurdsson

**Affiliations:** ^1^ New York University Grossman School of Medicine, New York, NY USA; ^2^ New York University, New York, NY USA

## Abstract

**Background:**

Abnormal neuronal activity was observed in awake and behaving tauopathy mice using two‐photon calcium imaging. Our previous study has revealed the relationship between tau pathology and altered neuronal calcium dynamics within the motor cortex of JNPL3 tauopathy mice at different stages of disease progression, specifically at 6 and 12 months of age (Wu Q et al, Neurobiol. Dis. 2021; Ji C et al, Alzheimers Dement 19, e074196; Ji C et al, Alzheimers Dement 18, e069415). However, network activity in tauopathy models remains less studied. Furthermore, the mechanisms responsible for the altered neuronal and network activity warrant further investigation.

**Methods:**

Somatic calcium activity in the motor cortex of 6‐month‐old homozygous JNPL3 mice and age‐matched wild‐type controls was imaged using two‐photon microscopy, when the animals were resting versus running. Neuronal Ca^2+^ activity profiles and synchrony were analyzed. To explore the mechanisms underlying the altered neuronal and network activity, neuronal excitability and synaptic transmission were assessed by electrophysiological recordings in acute brain slices.

**Results:**

We observed neuronal hypoactivity in the motor cortex of 6‐month‐old JNPL3 mice, when the animals were at rest or running on a treadmill. Neuronal Ca^2+^ activity exhibited enhanced synchrony and dysregulated responses to the running stimulus in JNPL3 mice. Furthermore, electrophysiological recordings in these motor cortical neurons of acute brain slices revealed a reduction in spontaneous excitatory synaptic transmission in pyramidal neurons and enhanced excitability of inhibitory neurons. These findings are likely linked to hypofunctional neurons and altered network activity in JNPL3 tauopathy mice.

**Conclusion:**

In the JNPL3 tauopathy mouse model, a reduction in neuronal Ca^2+^ activity was observed in the motor cortex of awake and behaving animals during the early stage of tauopathy. This neuronal deficit was more pronounced under running conditions. The presence of tau pathology affected the local neuronal circuitry, as evidenced by an increased basal Ca^2+^ activity synchrony and altered profiles of running‐related neuronal responses in the motor cortex. Underlying mechanisms may be linked to impairments in excitatory synaptic transmission and altered excitatory/inhibitory balance in this region, resulting from the accumulation of pathological tau.

